# Non-Cellular Layers of the Respiratory Tract: Protection against Pathogens and Target for Drug Delivery

**DOI:** 10.3390/pharmaceutics14050992

**Published:** 2022-05-05

**Authors:** Eleonore Fröhlich

**Affiliations:** 1 Center for Medical Research, Medical University of Graz, 8010 Graz, Austria; eleonore.froehlich@medunigraz.at; Tel.: +43-316-38573011; 2 Research Center Pharmaceutical Engineering GmbH, 8010 Graz, Austria

**Keywords:** epithelial barriers, respiratory tract, mucus, pulmonary surfactant, drug delivery, mucociliary clearance, mucopermeation, mucoadhesion

## Abstract

Epithelial barriers separate the human body from the environment to maintain homeostasis. Compared to the skin and gastrointestinal tract, the respiratory barrier is the thinnest and least protective. The properties of the epithelial cells (height, number of layers, intercellular junctions) and non-cellular layers, mucus in the conducting airways and surfactant in the respiratory parts determine the permeability of the barrier. The review focuses on the non-cellular layers and describes the architecture of the mucus and surfactant followed by interaction with gases and pathogens. While the penetration of gases into the respiratory tract is mainly determined by their hydrophobicity, pathogens use different mechanisms to invade the respiratory tract. Often, the combination of mucus adhesion and subsequent permeation of the mucus mesh is used. Similar mechanisms are also employed to improve drug delivery across the respiratory barrier. Depending on the payload and target region, various mucus-targeting delivery systems have been developed. It appears that the mucus-targeting strategy has to be selected according to the planned application.

## 1. Introduction

To maintain homeostasis, the human body possesses epithelial barriers with different permeability. All of them consist of epithelia with variable height, number of cell layers and tightness. Non-cellular layers provide additional protection against the invasion of toxicants. The skin possesses physical (hairs, desquamation), biological (microbiota), chemical (sweat) and biochemical (fatty acids) defense mechanisms. Main protective mechanisms of the gastrointestinal tract include peristalsis (vomiting, diarrhea), the shedding of intestinal crypt epithelial cells, saliva and mucus, microbiota, acidic pH and proteolytic enzymes. The respiratory tract uses sneezing and cough reflexes, air turbulences, mucus and ciliary action to prevent the invasion of gases and particles. In addition to these frequently mentioned portals of entry, epithelia, such as the cornea and urogenital tract, cover smaller parts of the human body [1].

The greater thickness of the epidermis (77–267 µm) compared to the lining epithelia of gastrointestinal and respiratory tract characterizes the skin as a protective barrier [2]. The epithelium at the beginning of the airways, trachea and bronchi, and the epithelium of the intestine measure only 50 µm, which suggests that they have both a protective and absorptive function [3,4]. While epithelial layers of the respiratory and gastrointestinal barrier at some locations have a similar thickness, the mucus layer measures 123–830 µm in the gastrointestinal tract and maximally 50 µm in the proximal airways [5,6]. The thickness of the epithelial barrier decreases further along the respiratory tract to 2 µm in the regions, where the exchange of gases takes place. Although the tightness of the epithelial layer limits the entry, non-cellular layers can determine the concentrations of inhaled compounds and particles at the epithelium.

Non-cellular layers represent an important defense against environmental gases, air-borne particles and pathogens as potential toxicants and a barrier for anesthetic gases and drug-containing aerosols. 

This review will highlight the role of non-cellular layers of the respiratory tract in contact with gases and pathogens (viruses, bacteria, fungi) and pharmaceutical strategies to penetrate these barriers for drug delivery. To estimate the chance for penetration of the respiratory barrier, it is important to familiarize with the properties of the non-cellular layer covering the different regions of the respiratory tract, mucus and surfactant, which will be described in the first part of the review. The second part provides the information, to which, parts of the respiratory tract gases and pathogens have access, and how they interact with the non-cellular barriers. In the third part, the strategies to overcome the respective non-cellular barriers (mainly the mucus barrier) for drug delivery will be discussed.

## 2. Organization of the Respiratory Tract

**Figure 1:**
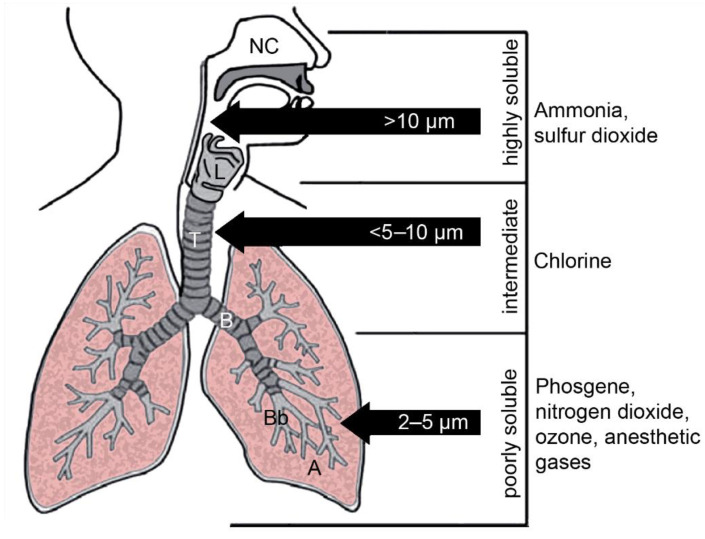
Upper respiratory tract with nose, nasal cavity (NC), mouth, pharynx and larynx (L), and lower respiratory tract with the (air) conducting parts trachea (T), bronchi (B) and bronchioles (Bb), and the respiratory part with respiratory bronchiole and alveoli (A), are shown. Contact of inhaled particles with the respiratory tract is mainly determined by size. Solubility in water regulates the action of gases in the airways. Examples for important gases with high, intermediate and poor water solubility are listed.

The larynx separates the upper respiratory tract from the lower respiratory tract. The upper respiratory tract also includes the nose, nasal cavity, mouth and pharynx (Figure 1). The trachea, the bronchi, bronchioles and alveoli are parts of the lower respiratory tract. The increase in the surface area from 40 cm^2^ (or 0.004 m^2^) in the trachea [7] to approximately 70 m^2^ in the alveolar part of the lung is the result of the specific architecture of the bronchial tree, which can be compared to a series of branching tubes [8]. The mouth and pharynx are common transit areas for the respiratory and gastrointestinal tract. The epithelium is similar to the lining of the oral cavity, which is mechanically more resistant than the respiratory epithelium. The nose and nasal cavity are only in contact with air and the epithelium resembles the coverage of trachea and bronchi. Measuring only 160 cm^2^ (or 0.016 m^2^), the nasal cavity plays an important role for drug administration because delivery to the brain, circumventing the blood–brain barrier, is possible [9].

### 2.1. Architecture and Rheological Properties of the Mucus Layer

**Figure 2:**
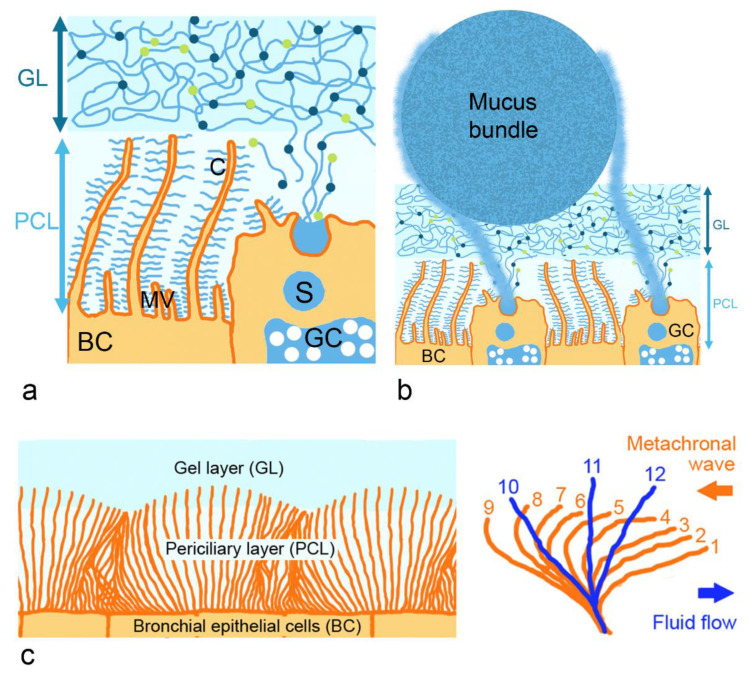
Respiratory mucus is arranged in periciliary (PCL) and gel layer (GL, (**a**)), where mucins form the structural basis of the mucus. Thick mucus bundles consisting of MUC5B and coated with MUC5AC nets, produced by the intraepithelial goblet cells (GC), are partly embedded in the GL (**b**). Mucus transport by ciliary beating relies on mucus gel layer and periciliary layer with correct viscosity and osmotic pressure (**c**). The tips of the cilia follow an elliptic trajectory during the beating. Abbreviations: BC, bronchial epithelial cells; C, cilia; MV, microvilli; S, secretory granule.

The epithelium of the conducting airways consists of bronchial epithelial cells with cilia and mucus-producing goblet cells (Figure 2a). Mucus, as the most prevalent non-cellular layer in this part, is 10–30 µm thick in the trachea and 2–5 µm in the bronchi [10]. Nasal mucus has a thickness of 10–15 µm [11]. In the trachea and bronchi, a periciliary layer surrounding motile cilia and a mucus gel layer on top of the periciliary layer can be discerned. The gel layer forms a network of fibers that act as a biological sieve, impeding the movement of particles [12]. Its main function is the trapping of particles and pathogens for subsequent removal by mucociliary clearance. By this clearance mechanism, particles are transported out of the bronchial tract to the pharynx and coughed out. Due to the density of the membrane-associated mucins in cilia and microvilli, only particles <40 nm can enter the periciliary layer [13]. 

Mucus consists of approximately 95% water, 0.2–5.0% mucins, 0.5% globular proteins, 0.5–1% salts and 1.2% lipids, DNA, cells and cellular debris. Important mucus features are the pH, pore size of the mesh, ionic strength and viscosity. Data for healthy and functional respiratory mucus have been reported as follows: pH of 6.5–7.9, irregular mesh size ranging from 100 nm to 500 nm, 165–211 mM sodium, 102–157 mM chloride, 13–17 mM potassium, 2.4–4 mM calcium, around 28 mM hydrogen carbonate and viscosity 0.04–10 Pa*s [12]. 

Viscoelasticity has to be high enough to prevent the mucus gel layer from sliding down due to gravitational forces. If it is, however, too high, cilia will not be able to transport the mucus. The beating of cilia is possible up to a fluid viscosity of 100 cP [14]. In addition, the osmotic pressure of the mucus gel layer is important for ciliary beating. It is higher (300 mPa) in the periciliary than in the mucus gel layer (200 mPa) [15]. This way, the gel layer is kept on top of the periciliary layer. The mucus gel layer represents a reservoir for water and can hydrate the periciliary layer to preserve ciliary beating required for mucociliary clearance. At a high salt and water concentration, the mucus gel layer swells [16]. At low salt and water concentrations, when the capacity of the gel layer to donate water to the periciliary layer is exhausted, the collapse of the mucus layer is seen. The mucin mesh of the mucus gel layer resembles a sieve restricting particle access by size and electrostatic exclusion. It appears that the surface of the gel mucus layer is not flat. According to a model published by Hansson [17], bundles of 1000–5000 MUC5B mucin linear polymers secreted by submucosal glands and coated by MUC5AC mucin nets produced by goblet cells are moved along the mucus gel surface by cilia (Figure 2b). The bundles are thicker than the mucus gel layer, move slower than the mucus and are arranged perpendicular to the direction of the flow. 

Membrane-associated mucins in the periciliary layer restrict the access to the epithelium and enable ciliary beating (Figure 2c). Mature bronchial epithelial cells contain up to 200 cilia with a density of 5–8 cilia/µm^2^ [18]. The movement includes retraction of the cilia upon bending in the periciliary layer and forward stroke in the extended form, in which, the tips of the cilia immerse into the mucus gel layer. Each cilium beats at the same frequency but in a phase-shifted manner with its neighbors along the axis of the effective stroke, and this results in metachronal waves. The trajectory of the cilium tip is assumed to follow an elliptic motion, and the beat velocity is around 12–15 Hz. The optimal thickness of the periciliary layer for the beating of the cilia is 6–8 µm. At a mucus solid of 15 wt%, the osmotic modulus of the gel layer starts to exceed that of the periciliary layer and the periciliary layer collapses. This leads to the decreased mucociliary clearance observed in cystic fibrosis patients [15]. Mucociliary clearance with approximately 20 min in the nasal cavity is fastest in the respiratory tract [19]. Velocity in the anterior part of the nose is negligible (1–2 mm/h), whereas, in the posterior part of the nose, 8–10 mm/min has been measured. Five mm/min indicates an average speed and was also obtained by computational fluid dynamic (CFD) simulations [20]. Mucus in the nasal cavity and in the bronchi have a similar composition and architecture. When terminal bronchioles become respiratory bronchioles (at generation 15–16 of the bronchial tract), the double-layered mucus structure with a gel layer and periciliary layer is replaced by a single mucus layer [21]. Epithelial cells lining the respiratory bronchioles lack cilia and mucociliary clearance is absent. Rheology at this point also changes because the lining fluid consists mainly of salt water plus the pulmonary surfactant [22].

#### 2.1.1. Gel-Forming Mucins

Gel-forming mucins form the structural basis of the mucin mesh and water is bound via their sugar residues. The main secreted mucins of the respiratory tract are MUC5B and MUC5AC. Both are large (5–50 MDa) heavily glycosylated proteins with characteristic structural domains [15]. MUC5B is predominant in the healthy airways and important for mucociliary clearance, whereas MUC5AC is upregulated upon viral infection and plays an important role in inflammation. MUC5AC has low mass/unit, contains small oligosaccharide units and has a stiff and extended conformation. MUC5B exists in high and low charged form and has a more compact conformation [23]. The levels of MUC5AC decrease along the bronchial tract, with highest levels in the trachea and lowest levels in the bronchioles [24]. The steeper decrease in MUC5B than in MUC5AC can be explained by the fact that MUC5AC is secreted by submucosal glands, which are not present in the bronchioles, whereas goblet cells in the bronchioles and club cells in the respiratory bronchioles maintain MUC5B production in the peripheral parts of the lung [25]. In the terminal bronchioles, there is neither MUC5AC nor MUC5B.

**Figure 3:**
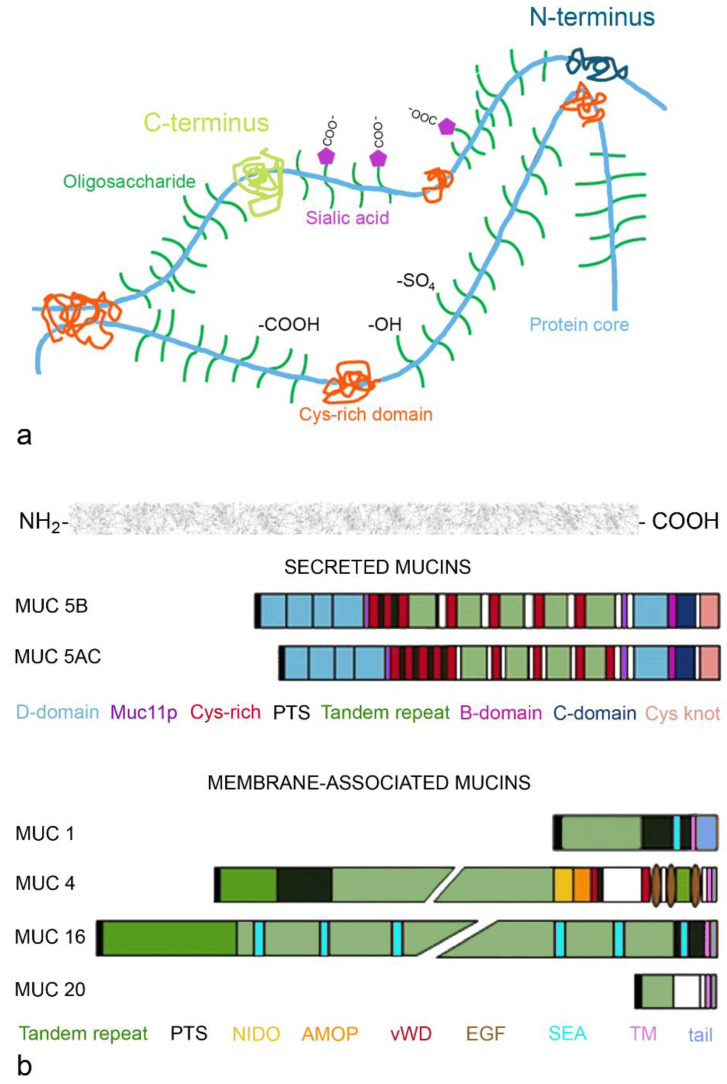
Mucin mesh (**a**) and structure of secreted and membrane-associated mucins (**b**). C- (yellow dots) and N-termini (blue dots) of the mucin monomers are sites of intermolecular disulfide bonds (**a**). Electrostatic interactions occur through carboxylic groups of sialic acid, hydrogen bonding through functional groups of the oligosaccharides and hydrophobic interactions via cysteine-rich domains. Secreted MUC5B and MUC5AC have D-domain at the C-terminus, and B-, C-domain and Cys knot are the von Willebrand factor-like domains important for polymerization (**b**). Domains of the membrane-associated MUC1, MUC4, MUC16 and MUC20 regulate cellular processes (growth, motility, differentiation). Abbreviations: AMOP, adhesion-associated domain in MUC4 and other proteins; EGF, epidermal growth factor; MUC11p, MUC11p15-type domain; NIDO, nidogen-like domain; PTS, proline, threonine, serine rich; SEA, sea urchin sperm protein; tail, cytoplasmic tail; TM transmembrane domain; vWD, von Willebrand factor D. MUC4 and MUC20 contain unique domains, which are shown in white.

Both MUC5B and MUC5AC can form mucin meshes, where C- and N-termini of the mucin monomers are sites of intermolecular disulfide bonds (Figure 3a). Electrostatic interactions occur through carboxylic groups of sialic acid, hydrogen bonding through functional groups of the oligosaccharides and hydrophobic interactions via cysteine-rich domains. The mesh-like structure is stabilized by repulsion of the negatively charged sulfate and sialic acid residues. Important parts of the mucin molecule include the von Willebrand factor-like domains at the N- and C-terminus for polymerization (D domain at the amino-terminus and B domain, C domain and Cys knot at the carboxy-terminus of the protein) and central tandem repeats (proline, serine and threonine (PTS)- rich repetitive and non-repetitive sequences; Figure 3b). The PTS parts carry the sugar residues—predominantly sialic acid for MUC5B and fucose for MUC5AC, which account for 80% of mucin weight. Highly conserved Cys-rich domains are interspersed and control the network via non-covalent cross-links between mucin polymers. 

#### 2.1.2. Membrane-Bound Mucins

Membrane-bound mucins include MUC1, expressed on the tips of the microvilli, and MUC4, MUC16 and MUC20, which are anchored in the membrane of the cilia [26]. All membrane-bound mucins contain extracellular O-glycosylated tandem repeats, the transmembrane domain and the intracellular cytoplasmic tail (Figure 3b). Membrane-bound mucins regulate physiologic functions of the epithelial cells. The cytoplasmic tail is important for intracellular signal transduction, while the extracellular glycosylated sites serve as recognition sites for glycan-binding proteins. The extracellular parts of the membrane-bound mucins differ in the number of sea urchin sperm protein endokinase and agrin domains (SEAs), which protect against mechanical damage and degradation. The longer MUC16 contains multiple, and MUC1 only contains one of these domains. The three epidermal growth factor (EGF)-like domains regulate the growth, motility and differentiation of the epithelial cells. The nidogen-like domain (NIDO) and adhesion-associated domain in MUC4 and other proteins (AMOP) are linked to cell adhesion, migration and angiogenesis. The shedding of mucus can occur at the SEA domains and may attract pathogens. MUC16 is also known as the tumor marker CEA125. The marker is, however, not specific for tumors involving the peritoneal cavity and can also be increased in inflammations involving the peritoneum (appendicitis, cholecystitis, salpingitis), endometriosis, hepatic cirrhosis and lung diseases [27]. 

### 2.2. The Pulmonary Surfactant Layer

Pulmonary surfactant is secreted by alveolar type II cells and non-ciliated bronchiolar cells (termed club cells) lining the terminal bronchioles. It is present mainly in the alveoli, but also in bronchioles and small airways, representing a continuum from small to large airways [28]. The main function of surfactant in the alveoli is the reduction in surface tension to forces near 0 mN/m at expiration, when the surfactant is compressed. In this situation, lipids at the surface form up to three layers and the bilayers under the surfactant monolayer increase the stability of the film and prevent the molecules from being squeezed out of the bilayer into the water phase. When the surfactant film is maximally compressed, forces of elastic tension and surface tension are balanced and there is no need for a further reduction in the alveolar surface or collapse [29]. 

Surfactant consists of 80% phosphatidylcholine (PC), of which, dipalmitoyl-PC (DPPC), palmitoyl-myristoyl-PC and palmitoyl-palmitoleoyl-PC together represent 75%. Anionic phosphatidylglycerol and cholesterol represent approximately 10% each, whereas the four surfactant proteins (SP)-A to -D comprise 2–5% [30]. SP-A represents the highest amount, with 4%, and SP-B and SP-C each make up less than 1% of the surfactant proteins. SP-A and SP-D are hydrophilic, whereas SP-B and SP-C are hydrophobic. DPPC plays a crucial role in the biophysical function, and anionic phospholipids, mainly phosphatidylglycerol, modulate the properties of the surfactant films. SP-B and SP-C contribute to the formation and stabilization of the surfactant films, while SP-A and SP-D, both members of the collectin family, interact with harmful agents and modulate the immune response. In general, SP-D acts more anti-inflammatory, whereas SP-A has both pro- and anti-inflammatory action. 

**Figure 4:**
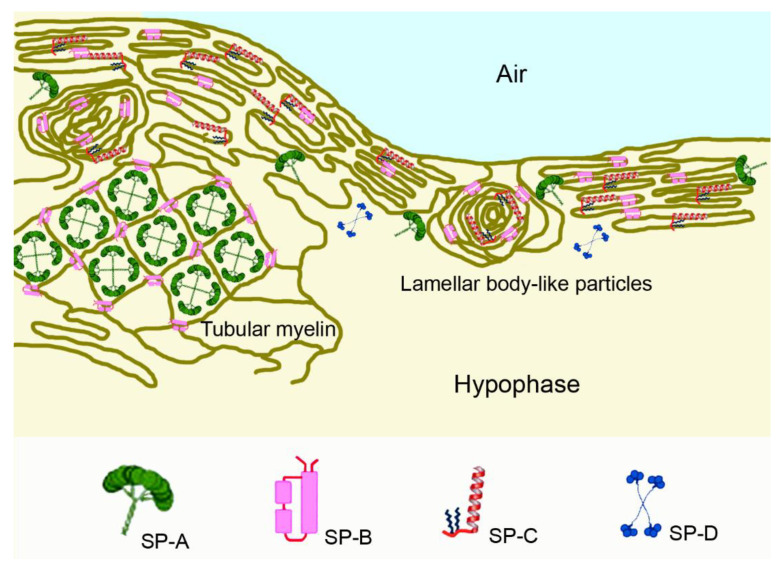
Pulmonary surfactant forms a lipid layer at the air–liquid interface above the hypophase (epithelial lining fluid) that covers the epithelial cell surface. Surfactant protein (SP)-B is important for formation and assembly of lamellar body-like particles, which can directly adsorb to the interface for surface film formation. Lamellar bodies secreted from alveolar epithelial type II cells (not shown in the figure) can unravel to form tubular myelin or rapidly move through a continuous network of surfactant membranes. During exhalation, the surfactant surface folds and SP-C plays a role in stabilizing the lipid membranes. Multilayered surfactant membranes form a reservoir for volume expansion of the alveolus upon inhalation. SP-A is integrated in a lattice of tubular myelin and binds simultaneously to different membranes, whereas SP-D exists as mainly soluble in the hypophase.

Lipids and proteins of pulmonary surfactant are assembled in alveolar type II cells and secreted into the hypophase (epithelial lining fluid) located between the epithelial surface and air as tubular myelin or as lamellar body-like particles (Figure 4). SP-B is necessary for the formation of tubular myelin and brings lateral stability to the DPPC-rich monolayer by electrostatic and hydrophobic interactions [31]. It promotes membrane–membrane contact formation in the compressed state and facilitates the formation of multilayered membrane arrays. SP-C contributes to the dynamic of surfactant films at the air–liquid interface by facilitating folding and preventing the expulsion of lipid/protein complexes from the interface upon compression. Upon expansion, it promotes the association of excluded surfactant structures; for instance, by the insertion of palmitoylated cysteine or protein into the tightly packed interfacial films [32]. Both proteins promote the insertion and re-spreading of phospholipids from the multilayers upon expansion. SP-A localizes to the corners of the tubular myelin lattice structure and modulates the mechanical properties of the surfactant film. SP-D is not localized to lamellar bodies or tubular myelin but binds weakly to the phospholipids of surface-active material and is mostly soluble in the hypophase [33]. Its role in the immune system consists of binding to influenza A and most probably also to other respiratory viruses, to components of Gram-positive and Gram-negative bacteria and to fungi [34]. 

## 3. Contact of Gases with the Non-Cellular Surfaces of the Lung

Access of gases to the respiratory tract, in principle, is less restricted than that of particles that can be filtered in the nose or deposited in the upper part of the tract due to their size. Inhalation of gases may occur accidentally at the workplace and as an air pollutant (sulfur dioxide). The extent of water solubility determines the region in which the gas can act. Examples of well-known gases differing in water solubility and with the (partly) identified mode of toxic action were chosen for illustration. As illustrated in Figure 1, highly water-soluble gases do not enter the bronchial tract, gases with intermediate water-solubility reach the tracheobronchial part of the respiratory tract and hydrophobic gases reach the distal parts of the respiratory tract. Toxic gases with different modes of action include ammonia, chlorine, nitrogen dioxide, phosgene and sulfur dioxide [35]. Highly water-soluble gases may not get into contact with smaller airways because they often have irritating and corrosive effects, resulting in reflex bronchoconstriction. Damage by the highly water-soluble ammonia is limited to the upper airways, where it dissolves into the mucus and produces ammonium hydroxide, a strong base [36]. It stays in the mucosa and acts locally corrosive and irritant. Only at a high concentration is ammonia systemically absorbed. Chlorine with intermediate water solubility primarily affects the upper airways, but also the lower airways. The formation of hypochloric acid and HCl in mucus contributes to its caustic and irritant effects in the conducting airways [37]. Contact of chlorine with the small airways led to surfactant dysfunction and altered the phospholipid content in the bronchoalveolar lavage fluid of treated mice [38]. Small airways collapsed and surfactant proteins SP-A and SP-D were oxidized. Nitrogen dioxide, phosgene and sulfur dioxide with poor water-solubility induced damage of bronchioles and alveoli. Phosgene rapidly dissolves in the surfactant and, by the generation of reactive oxygen species and lipid peroxidation, caused dysfunction [39]. The alteration of surfactant surface tension was also proposed as the main mechanism of toxic action in nitrogen-dioxide-induced pulmonary injury [40]. Similarly, sulfur dioxide caused the dysfunction of pulmonary surfactant after inhalation exposure of rats [41]. An increased secretion of phospholipids by alveolar type II cells was observed not only in nitrogen-dioxide-exposed rats but also in sulfur-dioxide-exposed rats, suggesting the induction of protective mechanisms [42]. It may be concluded that the dissolution of the hydrophobic gases consistently leads to surfactant dysfunction and that surfactant cannot prevent the penetration of hydrophobic gases into the respiratory tract. 

Exclusively gases with low water-solubility are used in general anesthesia. Halothane, isoflurane and sevoflurane diffuse across the mucus layer and may decrease mucociliary clearance by the inhibition of ciliary beating frequency. Their main targets are, however, the alveoli, where they cross the alveolar barrier to cause their anesthetic effects [43]. Their anesthetic action historically was explained by the Meyer–Overton rule [44]. According to this rule, potency is linearly correlated with the oil/water partition coefficient of the gas and was hypothesized to be caused by a change in orientation or solubility of proteins in the plasma membrane after integration of the gas molecules into the membrane. The interaction of hydrophobic gases with lipids could alter physiochemical properties of the membrane, or binding to membrane proteins could modulate membrane potential and ion transport. Halothane, enflurane and sevoflurane were able to decrease the cooperative interaction of phospholipids in the surfactant layer [45]. More recent studies identified various proteins as targets for anesthetic action, and the Meyer–Overton rule is currently no longer regarded as sufficient to explain the potency of anesthetic gases [46]. Studies also reported adverse effects of anesthetic gases, such as a decrease in the phospholipid synthesis of rat alveolar type II cells by halothane exposure. Exposure to O_2_ + desflurane and isoflurane increased levels of malondialdehyde, a marker for lipid peroxidation, in rat lung parenchyma [47]. The interaction of the anesthetic gases with the surfactant lipid monolayers leads to a decrease in their gel-to-liquid crystalline transition temperature. This bears the problem that, at a lower transition temperature, compression may not be enough to reach a sufficiently low surface tension [48]. An increased lysophosphatidylcholine and decreased phosphatidylcholine content in pulmonary surfactant and alveolar collapse were observed after the inhalation of sevoflurane by rats [49]. The authors concluded that the anesthetic promoted the fluidification of the condensed surfactant layers, which impaired the ability of the films to sustain the lowest surface tension. The adverse effect was reported in animals, but no clinical manifestations of pulmonary dysfunction have been observed in humans. There were even reports of potential beneficial effects of inhaled anesthetic gases on postoperative respiratory complications [50]. Different exposure conditions to the human situation (e.g., gas concentration, duration) and interspecies differences (e.g., lung architecture, cellular composition) may explain the observed differences.

## 4. Contact of Pathogens with Non-Cellular Surfaces

### 4.1. Infectious Aerosols

In contrast to gases, aerosols, defined as suspended particles in a gas or small droplets in air, affect respiratory regions depending on their size. For infection with viruses and bacteria, person-to-person transmission by aerosols is most relevant. Fungal infections are acquired by the inhalation of pathogens from the environment. 

For transmission from person to person, aerosol droplet sizes, generated during breathing, speaking, singing, coughing and sneezing, are relevant. Large droplets of diameters > 20 μm are too large to follow the inhalation airflow, and only smaller particles are regarded as inhalable [51]. Particles larger than 10 µm remain above the larynx, particles of sizes 5–10 µm deposit in the tracheobronchial part of the respiratory tract and only smaller particles reach bronchioles, respiratory bronchioles and alveoli (Figure 1). 

Studies reporting droplet size used different technologies and often included only few individuals. Therefore, different values are available in the literature. For instance for coughing, an average size of 8.35 µm, as well as ranges of 7–200 µm and 50–100 µm, have been reported [52]. When droplet sizes of cough and speech were compared, particles in speech aerosols were determined as larger than in cough aerosols (85.0 vs. 55.5 µm and 16.0 vs. 13.5 µm) [53,54]. Based on the available data, Gorbunov concluded that a size range from 1–100 µm for particles expelled upon sneezing and coughing appears realistic [55] and suggests that the deposition of pathogens occurs mainly in the conducting airways because droplets generated upon speech and coughing are too large. Once in the respiratory tract, viruses may reach deeper regions of the lungs after release from cells. 

### 4.2. Viruses

The most relevant viruses for severe respiratory illnesses are influenza A strains, respiratory syncytial virus (RSV) and the corona viruses severe acute respiratory syndrome coronavirus (SARS-CoV), middle east respiratory syndrome coronavirus (MERS) and SARS-CoV-2 [56]. Mucus is supposed to prevent infection by restricting the access and by the antiviral action of various proteins, such as β-defensin, lactoferrin, palate lung and nasal epithelium clone (PLUC), cathelicidins (LL-37), SP-A and SP-D, deleted in malignant brain tumor 1 (DMBT1) and galectins [13]. Rhinoviruses cause common but not severe respiratory effects. Viruses, on the other hand, may influence mucus properties [56]. Influenza A, respiratory viruses and RSV increase the viscosity and volume of the mucus gel layer. Influenza and RSV increase MUC5AC expression in A549 cells and the metapneumovirus and rhinovirus expression of MUC1, 2, 4 and 5AC in human bronchial epithelial cells. Mucus viscosity and retention are increased upon SARS-CoV-2 infection [13]. Due to the interaction with glycans, respiratory viruses, which usually possess capsids, do not easily cross the gel mucus layer [57]. Even without the interaction with glycans, influenza A viruses and corona viruses with a size of 80–120 nm are too large to penetrate the periciliary layer [58]. RSVs are even larger and present either as spheres of 100–350 nm diameter or filaments of up to 10 µm length and 60–200 nm diameter [59]. Only the small rhinoviruses (picornaviruses of 15–30 nm) could penetrate into the periciliary layer. They are non-enveloped viruses with single-stranded RNA surrounded by an icosahedral capsid consisting of three larger proteins and one smaller protein [60].

#### 4.2.1. Influenza Virus

The virus adheres to the mucus layer by the binding of hemagglutinin (HA) to sialic acid residues of the mucins [61]. Neuraminidase (NA) of the virus is able to cleave the acids and increases the permeation of the mucus layer. More invasive influenza A strains have a preference for sialylgalactosyl residues linked by 2–3 linkage, which are more common in the smaller airways [62]. Structural compounds and soluble factors act in concert to prevent viral infection. As structural components, MUC5AC inhibits influenza A replication. Lactoferrin, which acts as anti-viral and antibacterial, and nitric oxide can inhibit influenza A infections [63]. Antiviral action is species-specific and human mucus, not porcine mucus, inhibits influenza A H1N1 virus propagation. In the smaller airways, SP-D protects against influenza infection by interaction with mannose oligosaccharides located near the HA binding site of the virus, while SP-A directly occupies the HA-binding site. The exogenous administration of SP-A and SP-D can therefore protect against influenza A virus infections. However, if influenza virus strains have HA with little glycosylation, such as strains H1N1 (porcine) and H5N1 (avian), protection through SP-A and SP-D is not effective [62]. Palmitoyl-oleyl-phosphatidylglycerol also inhibited the invasion of various laboratory influenza A strains in vitro and in vivo [64].

#### 4.2.2. Corona Viruses

Spike, membrane and envelope proteins of the virus interact with mucins [65]. The corona viruses MERS-CoV, SARS-CoV and SARS-CoV-2 bind more to heparan sulfate of proteoglycans than to sialic acid, and the binding improves with the extent of the sulfatation of the heparan sulfate [66]. HA esterase removes sialic acids and facilitates mucopermeation, creating a high concentration of viruses near the cell surface. The contribution of mucins is not entirely clear. MUC4 expression was inversely linked to SARS-CoV-2 titer, suggesting a protective or neutralizing role of this mucin [67]. Since estrogen stimulates the transcription of MUC4, this finding may explain the fact that men have more serious instances of COVID-19 than women. High levels of MUC4, on the other hand, may have dehydrating effects on airway surfaces, leading to shortness of breath and worsening the pulmonary condition. High MUC1 and MUC5AC levels in sputum were linked to admittance to intensive care unit and high levels of shed mucus were linked to a worse outcome in COVID-19 patients. SP-D also binds to the spike protein and inhibits the cellular infection of HEK293T cells overexpressing human angiotensin converting enzyme 2 (ACE2), one of the receptors of SARS-CoV-2 [68].

#### 4.2.3. Respiratory Syncytial Virus

RSV has a virus envelope with highly glycosylated proteins, the attachment protein andthe fusion protein for ion channel formation to increase plasma membrane permeability [69], The small hydrophobic protein is also expressed by the virus and supposed to have ancillary function in virus-induced cell fusion. The virus lacks sialic acid cleaving capacity. Therefore, an increase in MUC5AC expression resulting in lowering mucopermeation is an effective defense mechanism against RSV infection [70]. Palmitoyl-oleoyl-phosphatidylglycerol and phosphatidylinositol, representing 10% of the pulmonary surfactant lipids, inhibit RSV by preventing the virus from binding to the epithelial cells [64].

### 4.3. Bacteria 

Bacterial infections of the upper respiratory tract are mainly caused by *Hemophilus influenzae*, *Streptococcus pneumoniae* and *Moraxella catarrhalis*, whereas *Pseudomonas aeruginosa* (35.3%), *Hemophilus influenzae* (33.8%), *Klebsiella pneumonia* (17.2%) and *Escherichia coli* (12.9%) affect the lower respiratory tract [71]. *Staphylococcus aureus* is rare in community-acquired pneumonias (1–10%) but present in 16% of hospital-acquired pneumonias [72]. The usual transmission of bacterial respiratory infections is via aerosol upon close contact with infected individuals. An exception is *E. coli*, which reaches the respiratory tract predominantly by the aspiration of oropharyngeal secretions or by hematogenous dissemination from a primary source in the gastrointestinal tract or the genitourinary tract [73]. Some bacteria, e.g., *S. pneumonia*, *M. catarrhalis* and *K. pneumonia*, are commensal, meaning that they exist in the healthy airways without causing adverse effects [74,75]. Cell adhesion and pro-inflammatory responses can be induced by exposure to cold temperature and cause bronchitis predominantly in patients with co-morbidities.

The trapping of bacteria in gel mucus is assumed to lead to inactivation of the pathogen and protection against invasion. Lactoferrin in respiratory mucus acts as antimicrobial and MUC1 specifically protects against infection with *P. aeruginosa* [63]. The size, shape and surface properties of the bacteria determine the ease with which they can cross the mucus gel layer. *H. influenzae* is one of the smallest bacteria, with a dimension of 0.3 × 1 µm, *S. aureus* and *S. pyrogenes* have diameters of 0.6–1 µm and *M. catarrhalis* has a diameter of 2–2.5 µm. *K. pneumoniae* is the relatively largest bacterium; it is a rod of 0.3–1 µm × 0.6–6.0 µm, whereas the other bacteria have a spherical to oval shape. Bacteria in general are hydrophilic [76] and have a negative surface charge [77], but differences may exist regarding bacterial wall properties, as *S. aureus* and *S. pyrogenes* are Gram-positive bacteria and *H. influenza*, *M. catarrhalis* and *K. pneumoniae* are Gram-negative bacteria. At first glance, one might think that, due to the higher content of hydrophobic molecules (lipids + lipoprotein (58% vs. 0–3%) and the lower levels of peptidoglycan (10–20% vs. 50%) than Gram-positive bacteria, Gram-negative bacteria are less hydrophilic [78]. However, the surface is coated with lipopolysaccharide (representing 13% of the bacterial wall), which consists of an inner hydrophobic region and a core polysaccharide with an O-specific polysaccharide at the outer part, providing hydrophilicity and a negative surface charge [79]. Peptidoglycan, rich in carboxyl and amino groups, and teichoic acids contribute to the negative charge of Gram-positive bacterial walls. This suggests no big differences in the surface hydrophobicity and charge between Gram-positive and Gram-negative bacteria, which is actually also seen in experiments. Similar behavior of Gram-positive and Gram-negative bacteria has been reported regarding the adhesion to positively and negatively charged methacrylate surfaces [80]. Both types of bacteria rapidly adhered to the positively charged surfaces, and only slowly adhered to the negatively charges surfaces. Therefore, it may be assumed that the retention of Gram-positive and Gram-negative bacteria in mucus is also similar.

It has been reported that bacteria cross freshly isolated mucus [81]. However, when mucus production is induced upon infection with bacteria, access to the epithelium is reduced or prevented. This protection is only temporarily beneficial and, usually, the thick mucus is removed when the infection is under control [17]. Patients with bronchitis experience that as coughing in the recovery phase, which serves to remove the excess mucus. If the accumulated mucus cannot be removed properly, as in chronic obstructive pulmonary disease or in cystic fibrosis, bacteria, particularly *P. aeruginosa*, use it as the basis for the formation of biofilms. The capability of this bacterium to degrade MUC5AC and MUC5B enables it to use the mucins as nutrients and to reach the epithelial cells despite the thick mucus layer [82].

The bacterial wall can also engage in hydrophobic interactions because the orientation of compounds is flexible. Relatively hydrophobic portions of protein or hydroxyl-deficient polysaccharide molecules can be differently oriented and render the surface more hydrophobic [79]. Interaction with small and large multilamellar vesicles of surfactant was demonstrated, and the vesicle binding of *H. influenzae* hindered the uptake by A549 cells [83]. In vivo, coating with surfactant decreased neutrophil recruitment and increased mucociliary clearance. SF-A is the best studied surfactant protein with anti-microbial action. It is hypothesized that the protein binds the lipopolysaccharide and bacteria by the lipid A moiety and enhances the uptake of bacteria by phagocytes [84]. SP-D, by contrast, appears to interact through core polysaccharide to inactivate lipopolysaccharide.

### 4.4. Fungi

Fungal infections of the lung are rare and comprise only 8–10% of nosocomial infections [85]. Patients with a compromised immune system, such as patients with acquired immune deficiency syndrome (AIDS) or cancer patients undergoing chemotherapy, as well as those patients who receive immunosuppressive therapy (e.g., in bone marrow/stem cell transplantation), may be affected [86]. The main species relevant for pulmonary infections are *Aspergillus* and *Cryptococcus* species. *Aspergillus* conidia can be inhaled and, due to their size of 2–3 µm, may reach the smaller airways and the alveoli. *Cryptococcus* species with sizes of 1–2 µm may reach the lung by inhalation of dust containing the yeasts. *Pneumocystis* species, such as *Pneumocystis jirovecii* (formerly *carinii*), are ubiquitously present in the environment and have also been found in healthy individuals. *Candida* pneumonia is a matter of debate because 50% of healthy individuals have lung colonization [85]. Generally, the conidia of *Aspergillus* species (*A. fumigatus* and *A. niger*) and not the trophic form of the fungus are inhaled. Conidia are asexual spores formed after mitosis and cytoplasmic cleavage. They contain an entire cell, in contrast to sexual spores (Ascospores, zygospores, oospores), which are formed after meiosis and are rarely seen in clinical isolates [87]. The composition of the inner part of all opportunistic fungi cell walls is similar and usually consists of chitin as the inner layer, followed by branched β-1,3-glucan and β-1,6-glucan layers [88]. The fungi contain hypermannosylated N- and O-linked glycans (mannans) in the outer layers. Wall compositions of relevant fungi are summarized in Table 1. It can be noted that conidia have a more hydrophobic surface than trophic *Aspergillus* forms, which is due to the presence of the hydrophobin rodlet layer of highly hydrophobic portions (hydrophobins) on the conidia [89]. With the exception of conidia, the surface carries hydrophilic charged groups, which can interact with the mucin glycans [90]. This binding can lead to the sequestration of the pathogens, but *Aspergillus* spp. possess proteases and glucosidases to degrade mucins and to use them as nutrients, which increases the permeability of the mucus barrier. 

SP-A and SP-D can agglutinate *Aspergillus fumigatus* conidia and increase the binding to alveolar macrophages [84]. Rat SP-A binds further to the major glycoprotein of *Pneumocystis jirovecii*. Decreased amounts of total lipids, phospholipids and phosphatidylcholine were seen in animal experiments and in patients. In combination with the increased levels of SP-A detected in the bronchoalveolar fluid of patients with pneumocystis infection, it was hypothesized that the pathogen uses lipids produced by the host for energy production [92]. In this case, pulmonary surfactant would not have a protective role and the substitution of the phospholipid may support pathogen proliferation. 

The defense of non-cellular layers against pathogens is mostly effectuated by mucus and consists of the anti-bacterial and anti-viral action of proteins and mechanical hindrance through the mucus mesh. Coating with surfactant proteins can decrease the uptake of epithelial cells and increase uptake by phagocytes, which results in a decreased pathogen load of the respiratory epithelial cells.

## 5. Overcoming Non-Cellular Barriers

As illustrated in the previous sections, mucus and surfactant play important roles in the protection of the lung against toxicants and pathogens. The respiratory system, however, is also a promising route for non-invasive drug delivery, and the non-cellular barriers decrease the efficacy of drug delivery systems. Most mucus-targeting formulations for the respiratory tract have been developed for nasal administration because the mucus layer is relatively thin (10–15 µm) [11]. This further allows nasal administration drug delivery from the nose to the brain, bypassing the poorly permeable blood–brain barrier. The mechanism by which compounds reach the brain from the nasal mucosa is not entirely clear. Transfer may be transcellular through either the sustentacular cells or the exposed olfactory sensory neurons of the olfactory bulb or via the trigeminal nerves [93]. Compared to products for oral inhalation, formulations for nasal administration are also easier to produce. Out of the different options for mucus-targeting formulations, the focus of this summary will be on particle-based formulations.

### 5.1. Mucus-Targeting Strategies

Binding to mucus to prolong persistence combined with an increased permeation to escape mucociliary clearance is used by many respiratory pathogens to increase the number of infected cells. Enhanced mucoadhesion or mucus penetration are also the strategies that are being used for drug delivery. Due to physiological differences, the most efficient strategy for drug delivery systems may differ between the gastrointestinal and respiratory tract. Mucus-bound particles or drugs in gastrointestinal mucus are cleared from the organism by mucus turnover, whereas they are removed by mucociliary clearance in the respiratory tract. These processes occur at different velocities. The removal of particles from the gastrointestinal tract has been indicated within 4–6 h in one study and 24–48 h in another [94,95]. By contrast, the gel layer of respiratory tract mucus is replaced every 10 to 20 min [96]. 

The selection of mucoadhesive or mucopermeating delivery should be decided based on the removal or renewal of the mucus at the epithelial barrier. Further, the type of release—extracellular, transcellular or intracellular—plays a role. In areas of fast mucus turnover, mucopermeating particles would be expected to be more efficient than mucoadhesive. Further, carriers functionalized with mucoadhesive polymers have problems in crossing the mucus layer. They are therefore not suitable for systemic release or intracellular delivery (e.g., gene therapy) [10]. It is suggested that delivery systems that aim for systemic delivery or intracellular delivery should use techniques based on mucopermeation rather than mucoadhesion. A recent study that compared the delivery of baicalin surrounded by a chitosan layer with and without coating with Pluronic^®^ F127 reported a higher efficacy of the mucus-permeating nanoparticles than of the mucus-adhesive nanoparticles in vitro and after aerosol inhalation by mice [97]. 

#### 5.1.1. Mucoadhesion

Mucoadhesion, defined as the attachment of a drug-loaded carrier to a biological membrane, involves the wetting, swelling, adsorption and interpenetration of the polymer chains [98]. Theories for mucoadhesion include wetting (the ability of the polymer to spread and develop intimate contact with the mucus membrane), attractive electronic effects (electrostatic forces between glycoprotein mucin network and bioadhesive material), adsorption (covalent, ionic and hydrogen bonds and van der Waals forces resulting in chemical bonding), diffusion (physical entanglement of mucins strands and flexible polymer chains) and fracture (relation of the forces for detachment related to the strength of adhesion). 

**Figure 5:**
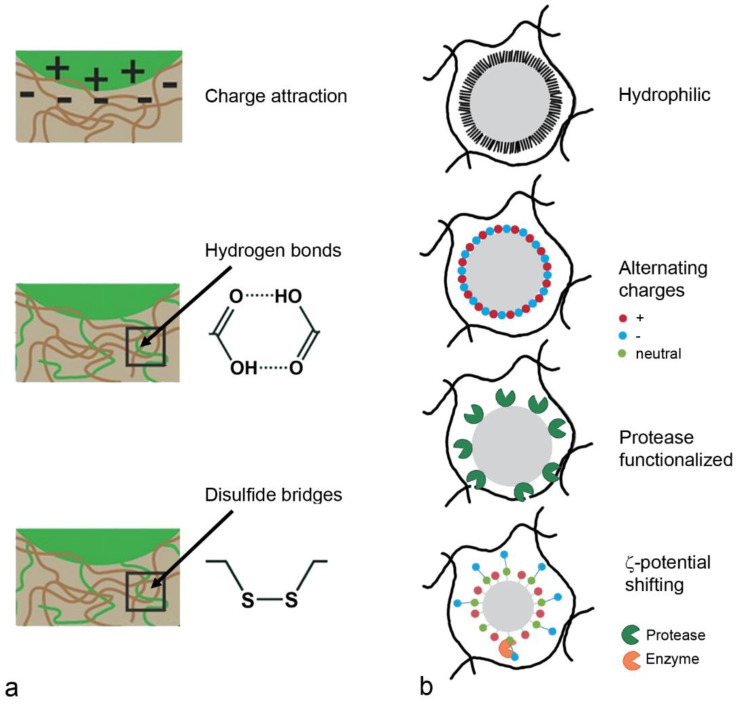
Coating for drug delivery systems based on mucoadhesion (**a**) use electrostatic interaction, hydrogen bonds and disulfide bridges. Mucopenetration (**b**) can be achieved by hydrophilic particles, particles with alternating charges, protease-functionalized particles and ξ–potential shifting particles. Colored dots indicate positive (red), negative (blue) and neutral (green) charge.

First generation mucoadhesive materials were synthetic hydrophobic molecules with numerous organic functions capable of forming hydrogen bonds, such as carboxyl, hydroxyl and amino groups [99]. These polymers, carbomers, chitosans, alginates and cellulose derivates adhere to mucus through different mechanisms (Figure 5a). Chitosan contains cationic groups that interact with the mucus surface due to the negative charge of mucins at neutral pH. Anionic polymers (carbomers/polyacrylates, alginate and carboxylmethylcellulose) adhere to mucus based on hydrophobic interactions and van der Waals bonds. Nonionic polymers, hydroxypropylmethylcellulose, hydroxyethylcellulose and methylcellulose use similar mechanisms but have weaker mucoadhesion compared to anionic polymers. Thiolated polymers, chitosan conjugated with thioglycolic acid, thiobutylaminidine, thioethylamidine or glutathione increase mucoadhesion due to the formation of disulfide bridges with cysteine residues from mucins [100]. In addition, thiomers promote mucopermeation and have anti-protease activity by the binding of divalent cations (Zn, Mg), which are important co-factors of many proteases [99]. Second generation systems are lectins (vegetal glycoproteins), which non-covalently bind to the glycosylated compound of the cell membrane, but not to the mucus layer.

Mucoadhesive polymers can be classified according to their source (natural/synthetic), solubility (water soluble/water insoluble) and charge (anionic, cationic, neutral) [101]. The molecular weight, flexibility, hydrogen-bonding capacity, crosslinking capacity, charge, polymer concentration and hydration/swelling determine mucoadhesion from the polymer side and pH, contact time, mucus turnover and disease state determine mucoadhesion from the biological side. This indicates that polymers may not be suitable for all administration routes.

#### 5.1.2. Mucopermeation

General rules have been developed for the passage of drugs across mucus. Size is an important factor, both for hydrophilic and for hydrophobic molecules, but the importance of charge is not completely clear [10]. In one study, positively charged, small drugs (tobramycin, gentamycin, amikacin) were most hindered by binding to mucins, whereas, in another study, the effect of charge was rather insignificant. Lipophilicity was assumed to have more importance than charge because interaction with glycoproteins and lipids takes place independent of the pH. It is expected that the mucus permeation of small molecules is less dependent on the hydrophobicity and surface charge than that of pathogens and carrier systems. Mucopermeation follows the strategy of rendering the carriers hydrophilic or generating a neutral surface charge in order to prevent electrostatic or hydrophobic interactions with mucus (Figure 5b). 

Polyethylene glycol (PEG), Pluronic^®^ polymers and polyvinyl acetate (PVA) are the most often used hydrophilic polymers used for mucopermeation. Coating improved the mucopermeation of carboxyl-modified polystyrene and poly(lactic-co-glycolic acid) (PLGA) particles [57]. The PEG-coated polystyrene and PLGA particles were evenly distributed along the mucosa, with an optimum at 10% PEG coating. PEGylated and Pluronic^®^ F127-coated 200 nm PLGA particles permeated freshly expectorated CF sputum significantly better than Pluronic^®^ F68-coated and non-coated particles. PEGylated PLGA particles were also better retained in mouse lungs 6 h after intranasal administration than the non-functionalized ones (75% vs. 50%) [102]. There are different advantages and disadvantages of the hydrophilic polymers [103]. PEG is the gold standard and Food and Drug Administration (FDA)-approved, but excretion from the organism and stability against oxidative degradation is limited. Poly(2-alkyl-2-oxazolines) are stable and better excreted but not approved by the FDA. PVA has a generally regarded as safe (GRAS) status and excellent surface active properties, but these properties are strongly dependent on the degree of deacylation. Poly(N-(2-hydroxypropyl)methacrylamide) is widely explored as a carrier for anticancer agents, but is non-biodegradable and expensive. Poly(2-hydroxyethylacrylate) is biocompatible but not soluble in water, and biomedical applications are currently lacking. 

Another strategy for generating mucopermeating carriers uses alternating surface charges. This strategy has been adopted from the coating of surfaces to reduce non-specific adsorption, mainly of unwanted organisms (antifouling) [104]. Zwitterionic materials containing positive and negative charged moieties resulting in a net neutral charge are promising because they cross the mucus layer, bind water and resist protein binding [57]. Opposite charges in carboxyl- and amine-functionalized nanoparticles disrupted the mucus barrier and improved the permeation of 200 nm polystyrene particles up to 4.5 times in experiments with gastric mucus. Dilauroylphosphatidylcholine (DLPC) contains a hydrophilic phosphatidylcholine headgroup and a hydrophobic dodecylic acid chain. It self-assembles on PLA particles, and the aggregation of the particles was reported as 20-fold lower than that of PVA-coated PLA particles.

Modification of the particle surface with proteases is another possibility for increasing mucopermeation. Papain and bromelain linked to polyacrylate increased penetration through porcine intestinal mucus 4.27 and 2.21-fold, respectively [105]. In comparison, the effect on the porcine intestinal mucus permeation of trypsin bound to PLGA particles was lower (two-fold increase compared to not-functionalized PLGA) than that of papain and bromelain (both three-fold increase) [106]. The main issue with these formulations is the long-term stability [103]. The use of protease-coated carriers in respiratory delivery may not be possible because peptides are preferred candidates for this route of administration and the separation of components of the carrier and the payload is different.

More recently, zeta potential shifting nanoparticles have been developed. They cross mucus as negatively charged carriers and, after the enzymatic cleavage of functional groups by plasma membrane-bound enzymes, become positively charged [107]. This facilitated the immobilization of the carriers near the epithelial plasma membrane. Often, phosphate groups are used, which are cleaved by the membrane-bound alkaline phosphatase to hydroxyl groups with neutral charge. After the cleavage, the positive charge of the remaining groups prevails. This strategy has not been used for delivery to the respiratory system.

Surfactants (e.g., sodium dodecylsulfate, Tween 80, poloxamer) have been tested for their ability to increase the diffusibility of nanoparticles in intestinal mucus. Although Tween 80 showed promising properties, its use is hindered by the fact that it is not an approved excipient for pulmonary administration [108]. The same applies for cationic and anionic (=catanionic) surfactant mixture composed of dodecyltrimethylammonium bromide and dioctylsulfosuccinate sodium salt, which were shown to increase the spreading of formulations in mucin and cystic fibrosis mucus [109]. When surfactant-containing formulations reach the alveoli, it cannot be excluded that they interact with pulmonary surfactant and cause pulmonary dysfunction. 

Permeation enhancers, which are used in oral and nasal drug delivery systems, e.g., bile acids, fatty acids, phospholipids, cationic polymers, cyclodextrins and tight junction modulators, are supposed to alter plasma membranes and intercellular junctions [110]. As they do not interact with mucus, their mechanisms will not be discussed further.

#### 5.1.3. Carriers for Nasal Drug Delivery

Despite the fast removal of the mucus layer, mucoadhesive particles are useful for nasal formulations because the mucus layer is only 10–15 µm thick and highly permeable [111]. Efficient transcellular delivery has been reported for mucoadhesive nasal formulations. They were developed mainly for the treatment of the neurodegenerative pathologies Alzheimer’s disease (AD) and Parkinson’s disease (PD), insomnia, psychotic disorders (schizophrenia, depression), epilepsy, chronic pain and nausea to take advantage of the nose-to-brain connection [112]. Other applications include herpes simplex virus, immunization and cancer [98]. Description of the composition of the individual formulations is beyond the aim of this review and only examples of common mucoadhesive and mucopermeating carriers are mentioned, with the respective indication given in brackets. Chitosan and chitosan derivatives are the most popular mucoadhesive polysaccharides. They have the advantage of also facilitating mucus permeation. Chitosan nanoparticles or nanoemulsions were used for the delivery of estradiol (AD), rivastigmine (AD), thymoquinone (AD), bromocriptine (PD), ropinirole (PD), rasagiline (PD), pramipexole (PD), curcumin (cancer) and tapentadol (chronic pain) [113]. Trimethylchitosan has the advantage over chitosan of having a better water solubility at physiological pH and being permanently positively charged. It was used for the delivery of Leu-encephaline (chronic pain), tizandidine (muscular pain), cyclobenzaprine (muscular pain), ropinirole (PD) and selegiline (depression). Coating with chitosan resulted in promising nanocarriers for PLGA particles loaded with chlorpromazine (schizophrenia), liposomes with ghrelin (cachexia) and insulin and solid lipid carriers with β-site amyloid precursor protein cleaving enzyme 1 (BACE1) siRNA (AD) [98]. Polycarbophil, a polymer derived from polyacrylic acid, was used as microemulsions for the delivery of zolmitroptan and sumatriptan (migraine), clonazepam (myoclonic seizures) and diazepam and lorazepam (insomnia). Other mucoadhesive formulations for intranasal drug delivery also showed an improved transmucosal delivery: maltodextrin-based phospholipid-coated nanoparticles (morphin), methacrylic co-polymer-functionalized poly(ε-caprolactone) nanocapsules (olanzapine), alginate nanoparticles (venlafaxine), β-cyclodextrin albumin nanoparticles (tacrine) and Delonix regia gum-coated nanolipid carriers (ondansetron). For the nose-to-brain delivery of peptides, 70–200 nm size functionalization with PEG, PEGylated surfactant, chitosan or targeting moieties using PLGA, PLA, liposomes and nanostructured lipid carriers showed the most promising results [114]. The administration of chitosan together with basic fibroblast growth factor as a liquid in one study increased brain concentrations to a similar extent (1.95 times) and was similarly efficient in encapsulation in gelatin-based nanocarriers reported in another study. The possibility of the co-delivery of more than one drug represents an obvious advantage of nanocarriers. Hydroxyl chitosan nanoparticles loaded with ketotifen (histamine release inhibitor) and decorated with cetirizine (histamine receptor antagonist) for the treatment of allergy reduced the treatment frequency to every 4 days and the dose to 50% in rats [115]. 

Further, nasal administration of mucoadhesive nanoparticles was successful for immunization against hepatitis B using chitosan and glycol chitosan-coated PLGA nanoparticles and chitosan-coated liposomes [98]. The oral treatment of high-risk patients with ivermectin proved to be ineffective in preventing the progression of coronavirus disease 2019 (COVID-19) infection to severe disease in a randomized controlled trial [116]. Nevertheless, it was reported that a mucoadhesive formulation consisting of the polymer mixture (hydroxypropylemthylcellulose 15,000, carbopol 974P and sodium alginate) and Poloxamer 407 and Poloxamer-188-stabilized ivermectin nanosuspensions resulted in a shorter duration of fever, cough, dyspnea and anosmia in a population of 114 patients, partly (41.2%) with co-morbidities [117]. 

Fewer mucopermeative polymers than mucoadhesive polymers have been used for nasal drug delivery. Sonvico et al. mentioned Pluronic^®^ F127 PLGA nanoparticles for diazepam and midazolam (epilepsy), tocopherol polyethylene glycol succinate (TPGS) micelles for zolmitriptin and sumatriptan (migraine), Lipid/PLGA nanoparticles for farnesylthiosalicylic acid (glioblastoma), poloxamer 188 cubasomes for olanzapine and spanlastics for risperidone (schizophrenia), gelatin-nanostructured lipid carriers for basic fibroblast growth factor (PD), polysorbate 80 solid lipid carriers for rosmarinic acid (Huntington’s disease) and novasomes for zolmitriptan (migraine). The authors pointed out that, in the reports on mucopermeating particles, the most important control, the liquid formulation plus the permeation enhancer, was lacking. It is therefore not possible to conclude that mucopermeating nanocarriers performed better than conventional formulations because the permeation enhancer alone might have caused the increase in the delivery. A head-to-head comparison of haloperidol (schizophrenia) as mucopermeating formulations, not mucopermeating formulations, and in liquid form, showed a small superiority of the PEGylated formulation. The non-PEGylated formulation contained cationic Eudragit and anionic Eudragit L100-55, whereas, for the PEGylated formulation, the PEGylated Eudragit L100-55 was used [118]. Effects in rats were better for both formulations compared to liquid haloperidol, but only in the first 20 min. 

The combination of mucoadhesion and mucopermeation for nasal drug delivery, mimicking the action of pathogens that first adhere to mucus and then use different strategies to cross the mucus layer, has also been reported. Meloxicam potassium against chronic pain was delivered by spray-dried nanoparticles of a combination of 2-hydroxypropyl-β-cyclodextrin and α-cyclodextrin, hyaluronic acid and poly(vinylalcohol) [119]. The particles showed an increased permeability across the nasal mucosa in vitro and ex vivo. 

Conclusions on the performance of mucus-targeting formulations are usually based on assessment in healthy mucus. However, pathology-induced changes in mucus properties may affect the efficacy of the delivery system. Due to pulmonary infections, changes in pH and ionic strength may occur. Data from quartz crystal microbalance with dissipation in mucus-coated sensors showed that a decrease in pH and increase in ionic strength induced softer mucus with more hydrophobic areas [120]. These changes may affect the permeation. Nanocrystals of the antimycotic C109 stabilized with TPGS and embedded in hydroxypropyl-β-cyclodextran were able to diffuse through cystic fibrosis artificial mucus containing mucin type II and salts [121]. Bacteria were not present, which may explain why the advantage of PEGylation in cystic fibrosis mucus was not seen in another study by the same group. To deliver siRNA pool against the nuclear factor-κB (siNFκB), a non-PEGylated (DPPC) or PEGylated 1,2-distearoyl-sn-glycero-3-phosphoethanolamine (DSPE)-PEG lipid shell was prepared [122]. The PEGylated particles did not better permeate cystic fibrosis sputum with polymicrobial colonization than the non-PEGylated carriers, whereas, on the other hand, the non-PEGylated constructs were ingested to a greater extent than the PEGylated carriers by Calu-3 and 16HBE14o- cells. It is expected that the PEGylation will not affect antimicrobial activity because PEGylated ofloxacin nanoparticles showed a higher uptake and efficacy than the non-PEGylated particles [123]. 

The COVID-19 pandemic stimulated the development of different preventive strategies. Inhalable formulations of so-called “nanocatchers” consisted of membranes isolated from HEK293T cells that were stably transfected with human angiotensin-converting enzyme II (ACE2) plasmid and were coated with hyaluronic acid to achieve mucoadhesion [124]. The nanocatchers were efficient in inhibiting pseudovirus infection in hACE2-expressing mice. The mucus-targeted approach is less advanced in clinical development than the delivery of siRNA siR-7, which was stabilized by locked nucleic acids, and formulated with peptide dendrimer KK-46 to enhance cellular uptake [125]. siR-7 targets the SARS-CoV-2 RNA-dependent RNA polymerase and efficiently reduced the virus load and lung inflammation in Syrian hamsters. The formulation, termed Mir-19, has been tested in phase 1 and phase 2 clinical trials in Russia and received national approval on 22 December 2021, and is now being deployed in Russian hospitals (https://forbetterscience.com/2021/12/28/russia-brings-peace-19/, assessed on 25 April 2022).

#### 5.1.4. Carriers for Pulmonary Drug Delivery

The majority of drug carriers for pulmonary delivery do not use mucus-targeting but rely on deposition in the peripheral lung due to the particle size. Strategies to increase the cellular uptake of liposomes and polymer-based particles for obstructive lung diseases, pneumonia and lung cancer have been reported [126]. Similar to nasal delivery, chitosan coating is the most commonly used strategy for mucus-targeting, with main indications of tuberculosis, lung cancer and cystic fibrosis [127]. The decoration of chitosan nanoparticles with hyaluronic acid was useful for the delivery of the radical scavenger ferulic acid for asthma treatment [128]. Silymarin/curcumin-loaded albumin nanoparticles coated with chitosan displayed anti-inflammatory effects in the oleic acid acute lung injury mouse model [129]. Anti-viral activity against SARS-CoV-2 was shown in vitro.

The combination of mucus- and macrophage-targeting is preferred for the treatment of tuberculosis. Mannose-containing polysaccharides such as guar gum or mannan, or functionalization with mannose, increase uptake by macrophages, whereas the potential of other coatings, such as galactose, β-glucan, N-acetylglycosamine and folic acid, is not clear [130]. A longer lung retention was observed for hydroxypropylmethylcellulose, alginate and guar gum nanoparticles loaded with rifampicin and isoniacid and for chitosan rifampicin nanocomposites. Mannosylated lysine conjugated alginate microspheres loaded with isoniacid were both longer retained in the lung and ingested to a greater extent by macrophages. The efficacy of rifabutin was increased by encapsulation in genipin-crosslinked ethylcellulose/chitosan particles. 

For the delivery of antibiotics to the lung, mucopermeating particles have mainly been studied. PEGylated PLA, lipid-coated PLGA, a combination of PLGA, PVA and chitosan, TPGS-PLGA, alginate lyase-coated PLGA and PLGA plus DNse I have been developed [126]. The majority of these particles have only been tested in vitro and not in animal studies. The mucus-inert TPGS-coated particles appear particularly promising because they avoid unspecific interactions with mucus and surfactant and accumulate in biofilms, where TPGS is cleaved by bacterial enzymes. Mucus permeation by amphiphilic particles is intended by the grafting of PLA (hydrophobic) and poly(2-methyl-2-oxazoline; hydrophilic) or poly(2-methyl-2-oxazine; hydrophobic) on a α,β-poly(N-2-hydroxyl)-D,L aspartamide backbone. They were loaded with the 5-lipoxygenase inhibitor Zileuton for asthma therapy, but their in vivo efficacy is not yet known [131]. It was reported that changing the surface properties of particles is not the only way to influence mucus permeation. Modification of the cholesterol ratio of liposomes changed mucus permeation in the way that low to medium membrane fluidity (low to medium cholesterol ratio) increased mucus permeation [132].

Mucus-permeating particles were further used for pulmonary vaccination. The coating of PLGA particles with N-acetyl cysteine markedly increased the mucopermeation of PLGA particles [133]. The loaded innate defense regulator peptide IDR-1018 permeated 4.1-fold better and was more efficient in the coated PLGA particles than in the non-coated PLGA particles. A mixture of poly(β-amino ester) and PEG-conjugated poly(β-amino ester) at an optimized ratio was used to compact ovalbumin-expressing plasmids to yield mucus-penetrating particles. The particles were ingested by dendritic cells, and the delivered toll-like receptor ligands p(I:C) and CpG induced strong pulmonary immunity [134].

## 6. Conclusions

The permeability of mucus depends on several parameters (e.g., particle size, hydrophobicity, charge), whereas hydrophobicity is the main factor for crossing the pulmonary surfactant layer. Mucus retains bacteria and fungi based on steric, hydrophobic and electrostatic interactions. Viruses are retained in the mucus mainly by adhesion to the glycosylation groups of the mucins. Pathogens differ in their ability to permeate the mucus mesh because some of them have mucus-degrading enzymes. The mechanisms used by pathogens for infection of the host, mucoadhesion and mucopermeation, are also the most important strategies to improve drug delivery across mucosae. A great variety of mucus-targeting particles have been described in the literature. They aim to improve the delivery of poorly water-soluble drugs, deliver peptides or small molecules or induce mucosal immunity. Requirements for delivery systems are dependent on the payload and on the properties of the non-cellular layer at the region of the respiratory tract intended for delivery. This may explain why no mucus-targeting strategy is suitable for all applications. 

## Figures and Tables

**Table 1:** Main compounds of the walls of opportunistic fungi listed from plasma membrane to outer surface.

Fungus	Composition	Reference
*Aspergillus*, trophic form	Chitin, β-1,3-glucan and β-1,6-glucan, glucomannan, galactosaminoglycan, glycosylated proteins	[89]
*Aspergillus* conidia	Chitin, β-1,3-glucan and β-1,6-glucan, melanin, rodlet layer	[88]
*Cryptococcus*	Chitin, β-1,3-glucan and β-1,6-glucan, mannans, glucuroxylomannan, galactoxylomannan	[88]
*Mucorales*	Chitin, β-1,3-glucan and β-1,6-glucan, melanin, extracellular polysaccharide, protein	[88]
*Pneumocystis* cysts	β-1,3-glucan and β-1,6-glucan, protein, mannans	[91]

## Data Availability

Not applicable.

## Abbreviations

ACE2	Angiotensin converting enzyme 2
AIDS	Acquired immune deficiency syndrome
AD	Alzheimer’s disease
AMOP	Adhesion-associated domain in MUC4 and other proteins
BACE1	β-site amyloid precursor protein cleaving enzyme 1
CFD	Computational fluid dynamic
COVID-19	Coronavirus disease 2019
DLPC	Dilauroylphosphatidylcholine
DMBT1	Deleted in malignant brain tumor 1
DPPC	Dipalmitoyl phosphatidylcholine
EGF	Epidermal growth factor
FDA	Drug administration
GRAS	Generally regarded as safe
HA	Hemagglutinin
LL-37	Cathelicidins
MERS	Middle east respiratory syndrome coronavirus
NA	Neuraminidase
NIDO	Nidogen-like domain
PC	Phosphatidylcholine
PD	Parkinson’s disease
PEG	Polyethylene glycol
PLA	Polylactic acid
PLGA	Poly(lactic-co-glycolic acid)
PLUC	Nasal epithelium clone
PTS	Proline, threonine, serine rich
PVA	Polyvinyl acetate
RSV	Respiratory syncytial virus
SARS-CoV	Severe acute respiratory syndrome corona virus
SEA	Sea urchin sperm protein
SP	Surfactant protein
TPGS	Tocopherol polyethylene glycol succinate
vWD	von Willebrand factor D
